# Distribution Patterns of Non-SARS-CoV-2 Respiratory Pathogens Among Patients With Influenza-Like Illness During the COVID-19 Pandemic: A Cross-Sectional Study From Bangladesh

**DOI:** 10.7759/cureus.113476

**Published:** 2026-07-27

**Authors:** Sharmin Sultana, Abu Taher, Nirvana Haque, S M Rashed Ul Islam, Md. Nazrul Islam, Afzalun Nessa

**Affiliations:** 1 Virology, Bangladesh Medical University, Dhaka, BGD; 2 Anesthesia, Analgesia and Intensive Care Medicine, Bangladesh Medical University, Dhaka, BGD; 3 Data Science and Business, York University, Toronto, CAN

**Keywords:** bangladesh, co-infection, multiplex pcr, pandemic, respiratory tract infections, rhinovirus, sars-cov-2, streptococcus pneumoniae

## Abstract

Background: During the COVID-19 pandemic, diagnostic efforts were heavily concentrated on SARS-CoV-2, leaving patients who tested negative with limited etiological workup despite continuing to present with influenza-like illness. This study aimed to identify and characterize the non-SARS-CoV-2 respiratory pathogens responsible for illness in this group at a tertiary care hospital in Bangladesh.

Methods: We conducted a descriptive cross-sectional study at the Department of Virology, Bangladesh Medical University (BMU), from July to December 2022, enrolling 165 adults with influenza-like symptoms who tested negative for SARS-CoV-2. Nasopharyngeal swabs were analyzed by multiplex real-time PCR (VIASURE Respiratory Panel III kit, CerTest Biotec, Zaragoza, Spain). Pathogen distribution was assessed using Chi-squared goodness-of-fit (GoF) as a global test, confirmed by exact Monte Carlo multinomial simulation (100,000 runs). Individual pathogens were compared using binomial exact tests with Benjamini-Hochberg (BH)-false discovery rate (FDR) correction. Co-infection independence was assessed with one-sided Fisher’s exact tests plus BH-FDR correction; effect sizes are reported as odds ratios (ORs) with 95% confidence intervals (CIs).

Results: Of 165 participants (mean age 34.8 ± 12.7 years; 61.8% male), 59 (35.8%; 95% CI: 28.8%-43.3%) had at least one pathogen, yielding 46 viral and 29 bacterial pathogen detections. Both distributions deviated significantly from uniformity (viral: χ²(6) = 48.35, p < 0.001; bacterial: χ²(2) = 20.14, p < 0.001). Human rhinovirus was the most statistically significant viral pathogen (21/46; 45.7%; BH-adjusted p < 0.001; z = +6.08), and *Streptococcus pneumoniae* the most significant bacterial pathogen (21/29; 72.4%; BH-adjusted p < 0.001; z = +4.46). Co-infections occurred in 15 patients (9.1%); no pair reached significance after BH-FDR correction, though rhinovirus-influenza B (OR = 5.41; 95% CI: 1.39-21.12; BH p = 0.087) and *S. pneumoniae*-*Haemophilus influenzae* (OR = 11.83; 95% CI: 1.85-75.65; BH p = 0.087) showed the strongest trends.

Conclusion: Non-SARS-CoV-2 respiratory pathogens like human rhinovirus and *S. pneumoniae* were among the causes of substantial illness in Bangladesh throughout the pandemic. Broad-spectrum multiplex PCR testing should be integrated into the clinical workup of SARS-CoV-2-negative patients with persistent respiratory symptoms.

## Introduction

Acute respiratory tract infections (ARTIs) rank among the most burdensome categories of infectious disease globally. Collectively, they account for an estimated 3.5 million deaths per year [1], and in South Asian countries including Bangladesh, they represent one of the most common reasons patients present to primary and tertiary care alike [2]. The etiological spectrum is broad, encompassing seasonal influenza A and B, respiratory syncytial virus (RSV), human rhinovirus, adenovirus, parainfluenza viruses, and the bacterial pathogens *Streptococcus pneumoniae*, *Haemophilus influenzae*, and *Moraxella catarrhalis*, among others [3,4]. Before the COVID-19 era, this landscape was reasonably well mapped, and clinical management was guided accordingly.

The arrival of SARS-CoV-2 in late 2019 changed the diagnostic picture profoundly. Fever, cough, sore throat, fatigue, and dyspnea symptoms shared by virtually every respiratory pathogen became reflexively attributed to COVID-19, and laboratory efforts were correspondingly concentrated on SARS-CoV-2 detection [5]. Patients who tested negative for SARS-CoV-2 were frequently discharged without further etiological investigation, even when their clinical presentation was indistinguishable from pre-pandemic respiratory infections [6]. This carries real risks: patients with influenza, rhinovirus infection, or pneumococcal disease may receive neither appropriate antiviral nor antibiotic therapy, while inappropriate empirical antibiotics are prescribed to others, contributing to antimicrobial resistance [7].

At Bangladesh Medical University (BMU), a preceding study documented that SARS-CoV-2 was the dominant pathogen among patients with influenza-like illness during the pandemic, followed by rhinovirus and adenovirus [8]. What happened among the larger group of SARS-CoV-2-negative patients remained uninvestigated. Multiplex real-time PCR now makes it technically feasible to test for 15 or more respiratory pathogens simultaneously from a single nasopharyngeal swab [9,10], and deploying this capability in SARS-CoV-2-negative patients provides a direct window into the overlooked respiratory pathogen burden.

This study aimed to identify and characterize the non-SARS-CoV-2 respiratory pathogens detected among patients presenting with influenza-like illness who tested negative for SARS-CoV-2. We also give careful attention to the statistical methods used to analyze pathogen distribution data, and we report effect sizes and multiple-comparison-corrected p-values throughout.

## Materials and methods

Study design and setting

This was a descriptive cross-sectional study conducted at the Department of Virology, BMU, Shahbag, Dhaka, over six months from July to December 2022. This period fell within the ongoing COVID-19 pandemic but after substantial population exposure and partial vaccination coverage, placing it in a distinct epidemiological phase from earlier pandemic waves.

Study population

We screened 400 consecutive patients presenting with influenza-like symptoms to the BMU Fever Clinic. Of the 400 patients, 235 did not meet the inclusion criteria and were excluded. The remaining 165 patients met all eligibility criteria and formed the final study population. Inclusion required age ≥ 18 years; presentation with one or more influenza-like symptoms (fever or chills, cough, sore throat, rhinorrhea, myalgia, headache, fatigue, chest tightness, or dyspnea) within seven days of symptom onset; and a confirmed negative SARS-CoV-2 result by real-time reverse transcription (RT)-PCR at the BMU COVID-19 Laboratory. Patients were excluded if SARS-CoV-2-positive, if they had severe immunosuppression or a serious co-existing illness likely to confound respiratory symptom attribution, if they declined consent, or if records were incomplete.

Sample collection and laboratory methods

Trained staff collected nasopharyngeal swabs using sterile synthetic fiber-tipped applicators, transferring each immediately into viral transport medium (VTM) and transporting samples to the virology laboratory within two hours under cold-chain conditions (2°C-8°C). Processing took place under Biosafety Level 2 (BSL-2) conditions. Nucleic acid extraction was performed using the MyCROBEE/croBEE 2.0 Universal Extraction Kit (Cytiva Europe GmbH, Freiburg im Breisgau, Germany) per the manufacturer’s protocol.

Pathogen detection used the VIASURE Respiratory Panel III Real-Time PCR Detection Kit (CerTest Biotec, Zaragoza, Spain) on a QuantStudio 5 platform (Thermo Fisher Scientific, Waltham, MA, USA). The panel simultaneously detects 22 respiratory pathogens like influenza A and B, RSV, human rhinovirus, human adenovirus, human metapneumovirus, human bocavirus, parainfluenza viruses types 1-4, human enterovirus, human coronaviruses (229E, NL63, OC43, and HKU1), *Chlamydophila pneumoniae*, *Mycoplasma pneumoniae*, *Legionella pneumophila*, *H. influenzae*, *S. pneumoniae*, and *M. catarrhalis*. Internal positive and negative controls were included in every run.

Statistical analysis

All statistical analyses were conducted in Python 3.12 (SciPy 1.11, Statsmodels 0.14; Python Software Foundation, Fredericksburg, VA, USA). Continuous variables are reported as mean ± standard deviation (SD); proportions as frequency and percentage with 95% Wilson confidence intervals (CIs).

Before applying Chi-squared goodness-of-fit (GoF) tests, we confirmed that the expected cell frequency rule-of-thumb (≥5 per cell) was met for both viral (expected = 6.57 per cell) and bacterial (expected = 9.67 per cell) groups. Both global p-values were additionally verified using exact Monte Carlo multinomial permutation tests (100,000 simulations), which yielded results fully consistent with the Chi-squared approximation. The Chi-squared GoF served as the global test of whether detected pathogens were uniformly distributed; when significant, adjusted standardized residuals (|z| > 1.96) identified which individual pathogens drove the deviation. The uniform distribution was selected as the null hypothesis because, in the absence of prior evidence favoring any specific pathogen in this SARS-CoV-2-negative population, equal prevalence across all detected pathogens represents the most conservative and assumption-free baseline against which to test observed frequencies.

Individual binomial exact tests were then conducted for each pathogen against the uniform null probability (p₀ = 1/k, where k = number of distinct pathogens detected). The binomial exact test was used rather than a normal approximation because several observed counts were small (n = 1-3); this test is exact and valid at any sample size. All p-values were adjusted using the Benjamini-Hochberg (BH) false discovery rate (FDR) procedure. A BH-adjusted p-value < 0.05 was considered statistically significant.

For co-infections, the relevant question is not whether co-infection types are evenly distributed, but whether any two pathogens co-occur more than expected given their individual prevalences. We used one-sided Fisher’s exact tests on 2 × 2 contingency tables for each pair. Fisher’s exact test was chosen because it is an exact method valid regardless of sample size, requiring no minimum cell frequency. p-values were corrected using BH-FDR across all seven pairs. Effect sizes are reported as odds ratios (ORs) with 95% CIs calculated via the log-transformed Woolf method.

Ethical considerations

The study was approved by the Institutional Review Board of BMU (Ref. No. BSMMU/2021/12077, dated November 29, 2021). Written informed consent was obtained from all participants prior to enrolment. The study was conducted in accordance with the principles of the Declaration of Helsinki and applicable Good Clinical Practice guidelines.

## Results

Demographic and clinical characteristics

The 165 enrolled participants had a mean age of 34.8 ± 12.7 years (range: 18-72 years). The 18-30 age group was the largest (38.2%), followed by 31-40 years (26.1%), 41-50 years (19.4%), and over 50 years (16.4%). Men comprised 61.8% of the cohort; 58.2% of participants were urban residents. The mean duration of symptoms before presentation was 4.3 ± 2.1 days (Table 1).

**Table 1:** Demographic characteristics of study participants (N = 165). Data expressed as frequency and percentage or mean ± SD. SD: standard deviation

Variable	n (%) or mean ± SD
Age (years)	
Mean ± SD	34.8 ± 12.7
Range	18-72
Age group (years)	
18-30	63 (38.2)
31-40	43 (26.1)
41-50	32 (19.4)
>50	27 (16.4)
Sex	
Male	102 (61.8)
Female	63 (38.2)
Residence	
Urban	96 (58.2)
Rural	69 (41.8)
Symptom duration before presentation (days)	4.3 ± 2.1

Cough was the most common symptom, present in 74.0% of participants, followed by fever (67.9%), sore throat (52.1%), and rhinorrhea (49.1%). Systemic symptoms including myalgia (27.9%), fatigue (24.8%), and headache (21.8%) were present in roughly one-quarter of patients. Dyspnea was less common (6.1%), and gastrointestinal symptoms were infrequent (Table 2).

**Table 2:** Clinical symptoms reported by study participants (N = 165). Data expressed as frequency and percentage.

Symptom	n (%)
Cough	122 (74.0)
Fever	112 (67.9)
Sore throat	86 (52.1)
Rhinorrhea (runny nose)	81 (49.1)
Myalgia (muscle pain)	46 (27.9)
Fatigue	41 (24.8)
Headache	36 (21.8)
Dyspnea (shortness of breath)	10 (6.1)
Vomiting	8 (4.8)
Diarrhea	7 (4.2)

Overall pathogen positivity

**Figure 1:**
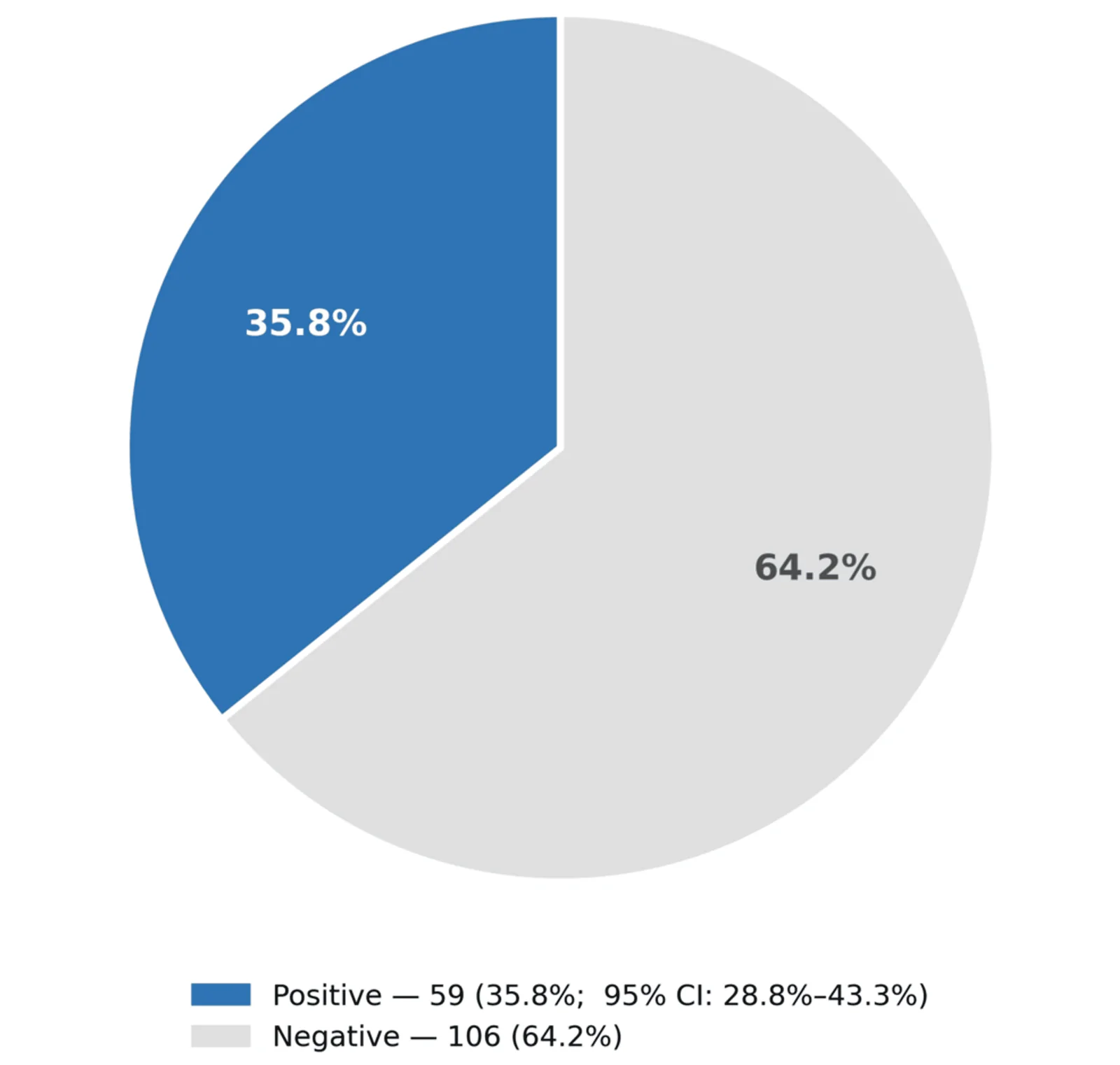
Overall pathogen positivity among study participants (N = 165). CI: confidence interval

**Figure 2:**
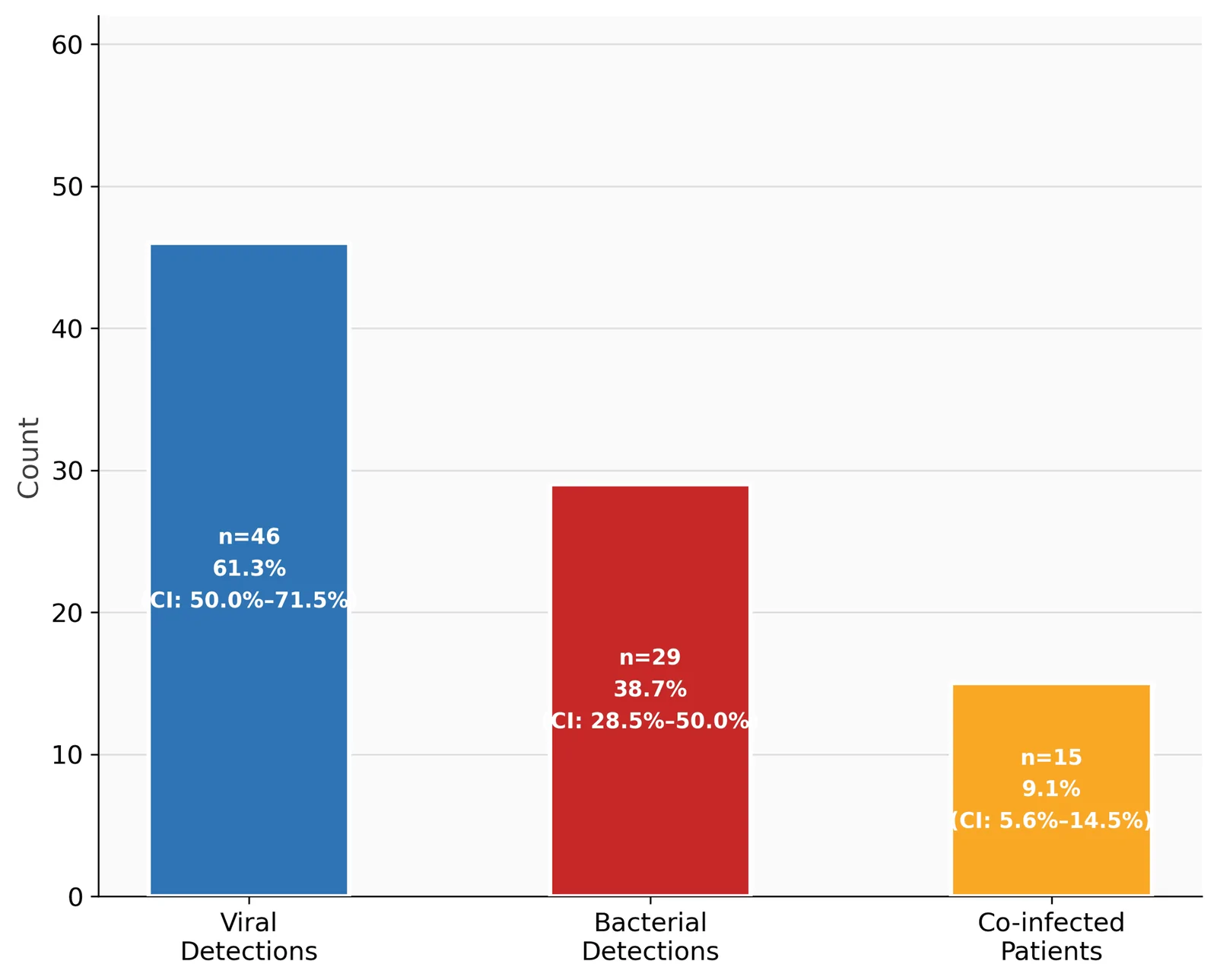
Breakdown of pathogen detections across 59 positive patients. Viral detections accounted for 61.3%, bacterial for 38.7%, and co-infections occurred in 9.1% of the enrolled cohort (95% CIs shown within bars). CI: confidence interval

Of 165 participants, 59 (35.8%; 95% CI: 28.8%-43.3%) tested positive for at least one respiratory pathogen. These 59 positive patients yielded 75 total pathogen detections: 46 viral and 29 bacterial. Viral detections accounted for 61.3% of all detections (95% CI: 50.0%-71.5%) and bacterial detections for 38.7% (95% CI: 28.5%-50.0%). Co-infections occurred in 15 (9.1%) patients of all enrolled participants (95% CI: 5.6%-14.5%). The overall breakdown is illustrated in Figures 1, 2.

Most significant viral pathogens

Seven distinct viruses were identified across the 46 viral detections. The global Chi-squared GoF test demonstrated that the distribution was far from uniform (χ²(6) = 48.35, p < 0.001; confirmed by exact Monte Carlo simulation, p < 0.001). Human rhinovirus was by far the most statistically significant and most frequently detected viral pathogen, accounting for 21 of 46 viral detections (45.7%). Its adjusted standardized residual of z = +6.08 and BH-adjusted p < 0.001 indicate unambiguous overrepresentation; it appeared at nearly three times the frequency of the next most common virus. Almost half of all virally infected patients in this cohort had rhinovirus. No other viral pathogen was significantly overrepresented after BH-FDR correction.

**Figure 3:**
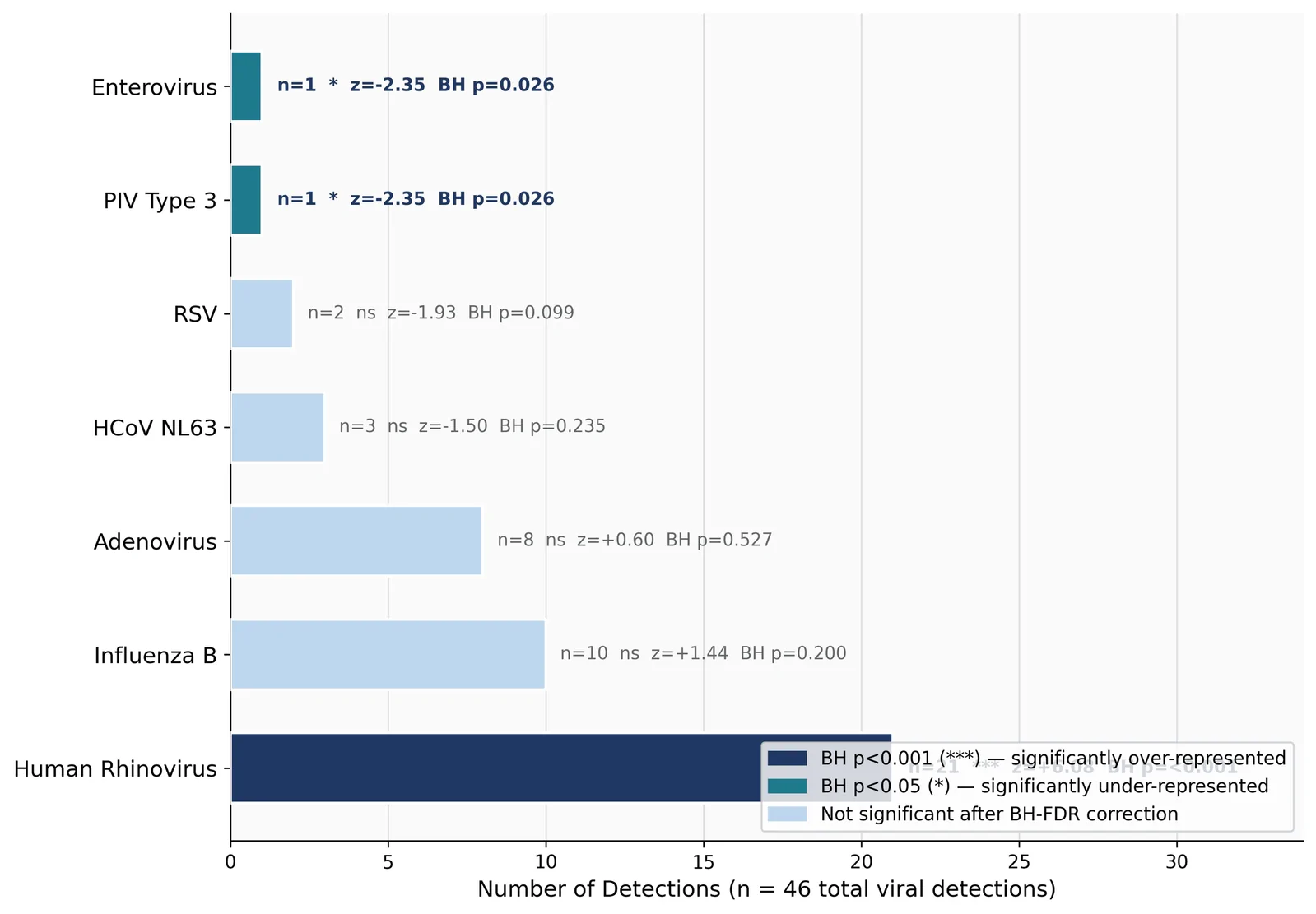
Viral pathogen distribution with BH-FDR-corrected significance. Human rhinovirus was the only significantly overrepresented pathogen (***BH p < 0.001; z = +6.08). Parainfluenza (PIV) type 3 and enterovirus were significantly underrepresented (*BH p = 0.026). Bars colored by significance status. BH: Benjamini-Hochberg; FDR: false discovery rate; RSV: respiratory syncytial virus

Parainfluenza virus type 3 and enterovirus were each detected only once (2.2% each) and were significantly underrepresented relative to the uniform null (z = −2.35 each; BH-adjusted p = 0.026), indicating their virtual absence from this cohort during the study period. Influenza B (21.7%), adenovirus (17.4%), HCoV NL63 (6.5%), and RSV (4.3%) were detected at intermediate frequencies but did not reach significance after correction. All results with standardized residuals and corrected p-values are in Table 3 and Figure 3.

**Table 3:** Distribution of detected respiratory pathogens with full statistical analysis. Exp.: expected count under uniform null (n/k); Adj. z: adjusted standardized residual; |z| > 1.96 indicates significant over-/underrepresentation; BH p: Benjamini-Hochberg FDR-corrected p-value; GoF: goodness-of-fit; FDR: false discovery rate ***BH p < 0.001 *BH p < 0.05 ^ns^Not significant after correction ^†^Significantly overrepresented ^‡^Significantly underrepresented. Individual tests by binomial exact test

Group	Pathogen	n (%)	Exp.	Adj. z	Raw p	BH p
Viral pathogens-46 detections, 7 viruses (global GoF: χ²(6) = 48.35, p < 0.001)						
Viral	Human rhinovirus^†^	21 (45.7)	6.6	+6.08	<0.001	<0.001***
	Influenza B virus	10 (21.7)	6.6	+1.44	0.143	0.200^ns^
	Human adenovirus	8 (17.4)	6.6	+0.60	0.527	0.527^ns^
	Human coronavirus NL63	3 (6.5)	6.6	−1.50	0.202	0.235^ns^
	Respiratory syncytial virus	2 (4.3)	6.6	−1.93	0.056	0.099^ns^
	Parainfluenza virus type 3^‡^	1 (2.2)	6.6	−2.35	0.011	0.026*
	Human enterovirus^‡^	1 (2.2)	6.6	−2.35	0.011	0.026*
Bacterial pathogens-29 detections, 3 organisms (global GoF: χ²(2) = 20.14, p < 0.001)						
Bacterial	*Streptococcus pneumoniae*^†^	21 (72.4)	9.7	+4.46	<0.001	<0.001***
	Haemophilus influenzae	5 (17.2)	9.7	−1.84	0.076	0.076^ns^
	*Moraxella catarrhalis*^‡^	3 (10.3)	9.7	−2.63	0.009	0.014*

Most significant bacterial pathogen

*S. pneumoniae* was overwhelmingly the most significant bacterial pathogen, accounting for 21 of 29 bacterial detections (72.4%; z = +4.46; BH-adjusted p < 0.001). The bacterial distribution as a whole deviated significantly from uniformity (χ²(2) = 20.14, p < 0.001). Nearly three-quarters of all bacterial respiratory infections identified in this study were caused by *S. pneumoniae*.

**Figure 4:**
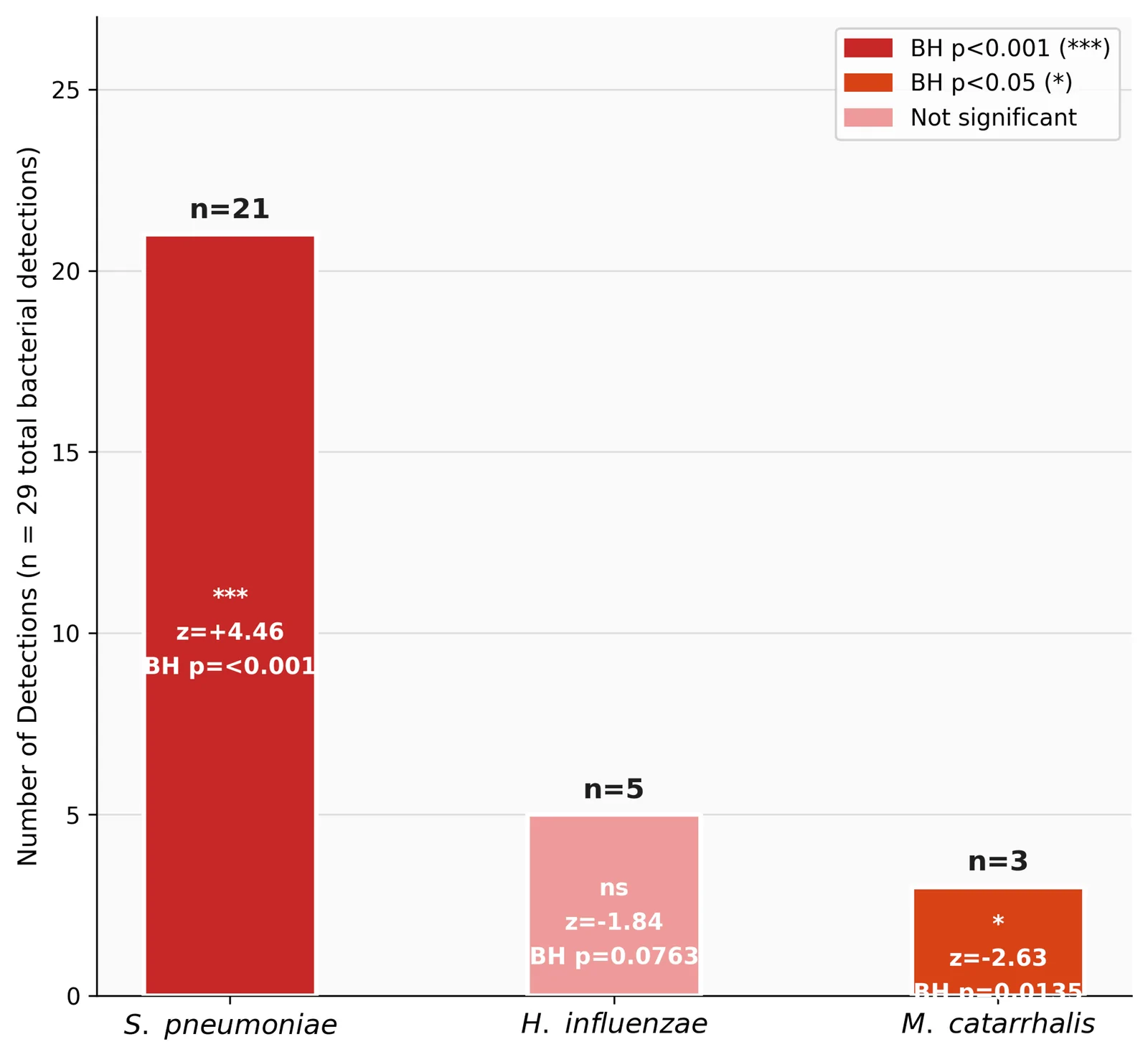
Bacterial pathogen distribution with BH-FDR-corrected significance. *Streptococcus pneumoniae* was overwhelmingly dominant (***BH p < 0.001; z = +4.46). *Moraxella catarrhalis* was significantly underrepresented (*BH p = 0.014). BH: Benjamini-Hochberg; FDR: false discovery rate

*M. catarrhalis* was significantly underrepresented (3/29; 10.3%; z = −2.63; BH-adjusted p = 0.014), while *H. influenzae* (5/29; 17.2%) did not reach significance after correction (BH p = 0.076). Full bacterial data are presented in Table 3 and Figure 4.

Co-infection analysis

Fifteen participants (9.1%; 95% CI: 5.6%-14.5%) had two pathogens detected simultaneously. The seven observed combinations are shown in Table 4. Fisher’s exact tests (one-sided) with BH-FDR correction found no co-infection pair statistically significant after correction (all BH p > 0.05). Two pairs showed the strongest trends toward non-random co-occurrence: (1) rhinovirus + influenza B: 4 observed vs. 1.27 expected under independence; OR = 5.41 (95% CI: 1.39-21.12); BH-adjusted p = 0.087; (2) *S. pneumoniae* + *H. influenzae*: 3 observed vs. 0.64 expected; OR = 11.83 (95% CI: 1.85-75.65); BH-adjusted p = 0.087.

**Table 4:** Co-infection patterns: Fisher’s exact test for statistical independence. Obs.: observed co-occurrences; Exp.*: expected under statistical independence (nA × nB/N); OR: odds ratio with 95% CI by log-transformed Woolf method. Fisher’s exact test one-sided (H₁: greater than expected). BH p: Benjamini-Hochberg FDR-corrected p-value. No pair significant after correction. N = 165. FDR: false discovery rate; CI: confidence interval

Co-infection pair	Obs.	Exp.*	OR	95% CI	Raw p	BH p
Rhinovirus + influenza B	4	1.27	5.41	1.39-21.12	0.025	0.087
Rhinovirus + adenovirus	1	1.02	0.98	0.11-8.38	0.672	0.754
Rhinovirus + *S. pneumoniae*	4	2.67	1.76	0.53-5.84	0.267	0.590
Adenovirus + *S. pneumoniae*	1	1.02	0.98	0.11-8.38	0.672	0.754
Influenza B + *S. pneumoniae*	1	1.27	0.75	0.09-6.24	0.754	0.754
*S. pneumoniae* + *H. influenzae*	3	0.64	11.83	1.85-75.65	0.015	0.087
*S. pneumoniae* + *M. catarrhalis*	1	0.38	3.55	0.31-40.96	0.337	0.590

**Figure 5:**
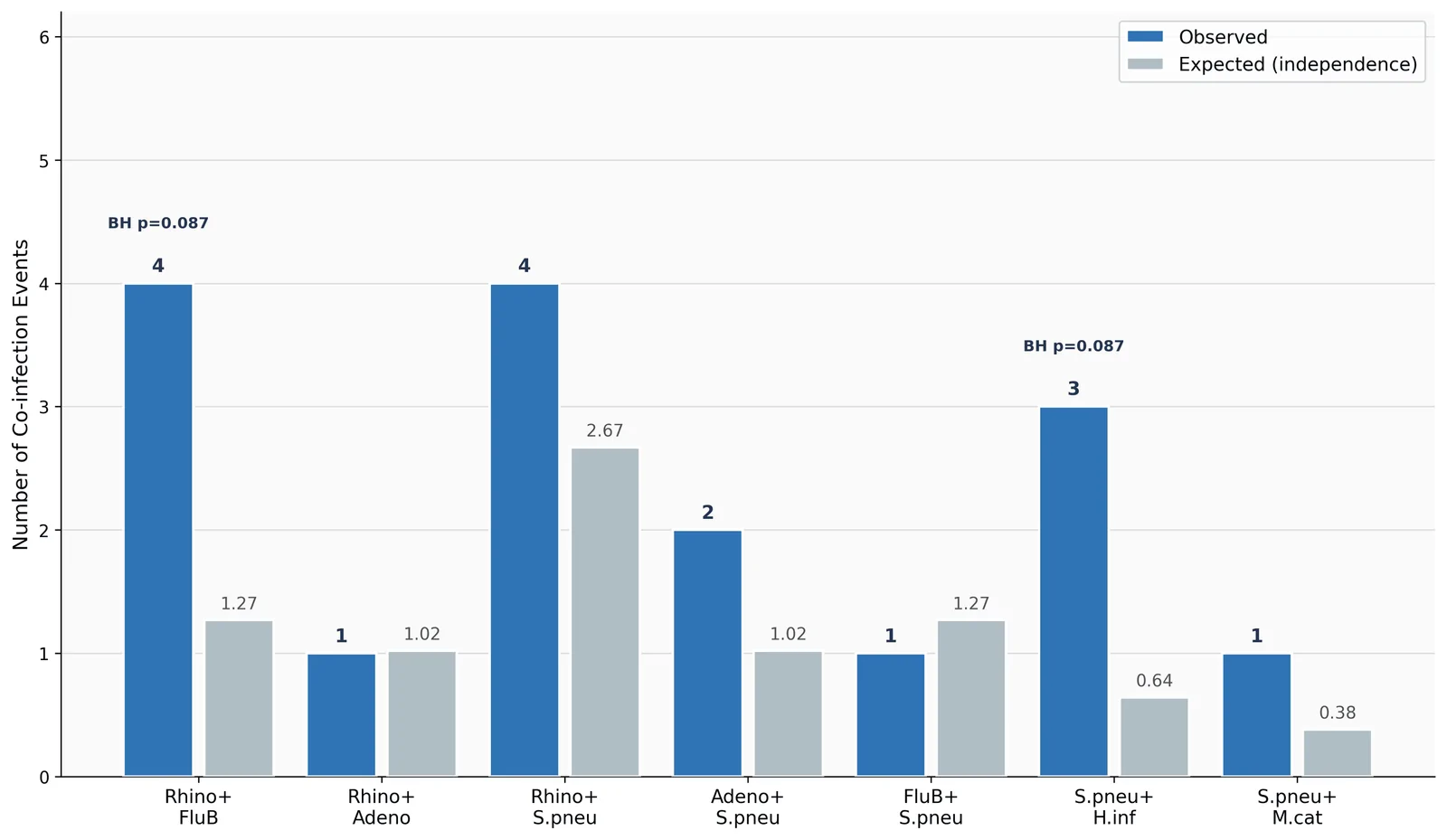
Co-infection patterns: observed versus expected counts under statistical independence (Exp.* = nA × nB/N). Pairs whose observed bar exceeds expected show a trend toward non-random co-occurrence. BH-adjusted p-values shown for pairs with BH p < 0.10. No pair reached significance after BH-FDR correction. BH: Benjamini-Hochberg; FDR: false discovery rate

**Figure 6:**
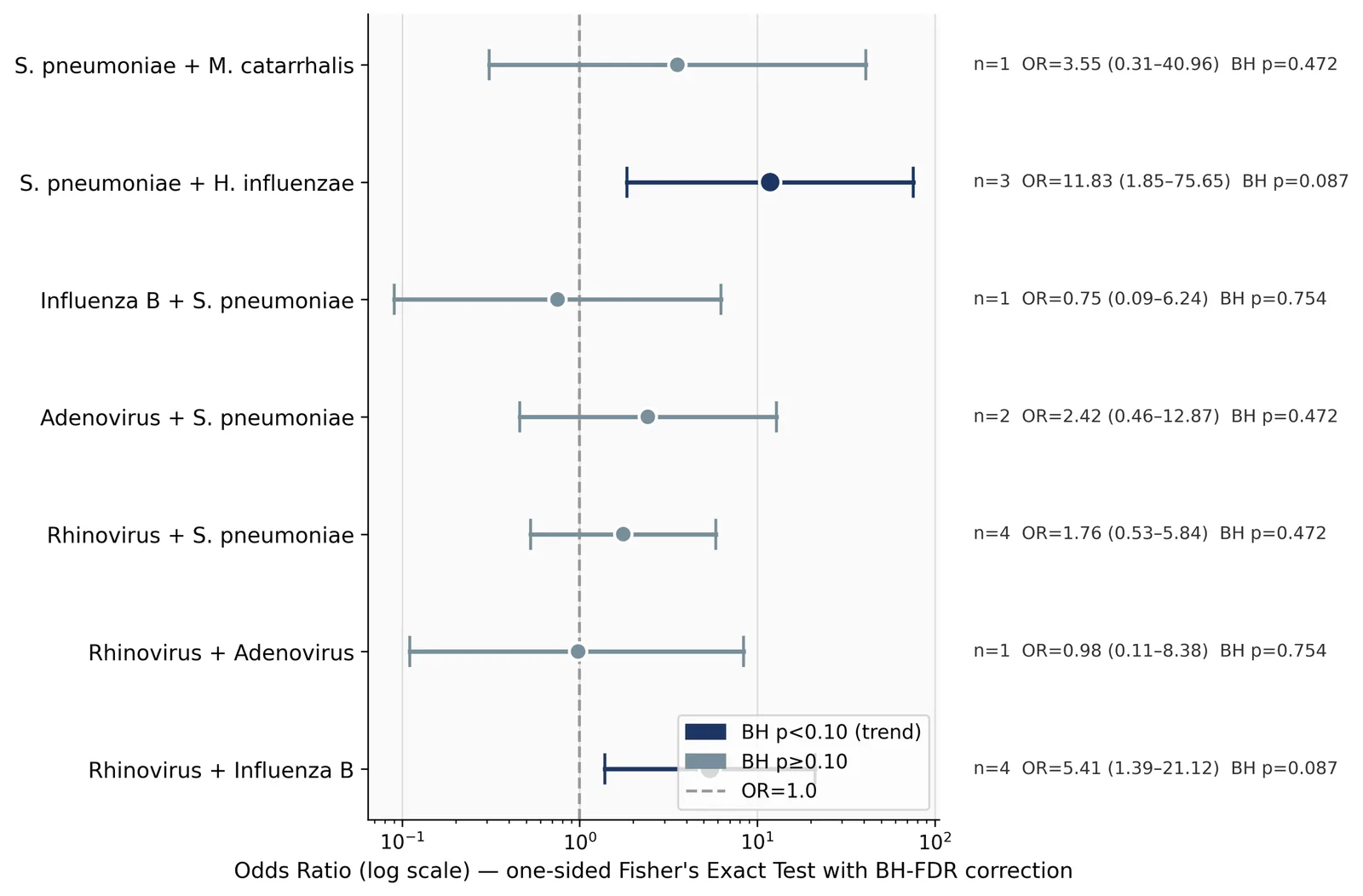
Forest plot of odds ratios (ORs) (log scale) for all seven co-infection pairs with 95% confidence intervals. The dashed vertical line represents OR = 1.0 (no association). Navy points indicate pairs with BH p < 0.10. Wide confidence intervals reflect the small number of co-infection events. No pair is statistically significant after BH-FDR correction. BH: Benjamini-Hochberg; FDR: false discovery rate

Both pairs mentioned above fall just short of the corrected significance threshold. The wide CIs are consistent with the small number of co-infection events and indicate that while the point estimates are suggestive of potential biological associations, the current data cannot support definitive conclusions. These findings are best treated as trends warranting investigation in a larger cohort. Observed versus expected counts and a forest plot of ORs are shown in Figures 5, 6.

Seasonal distribution

**Figure 7:**
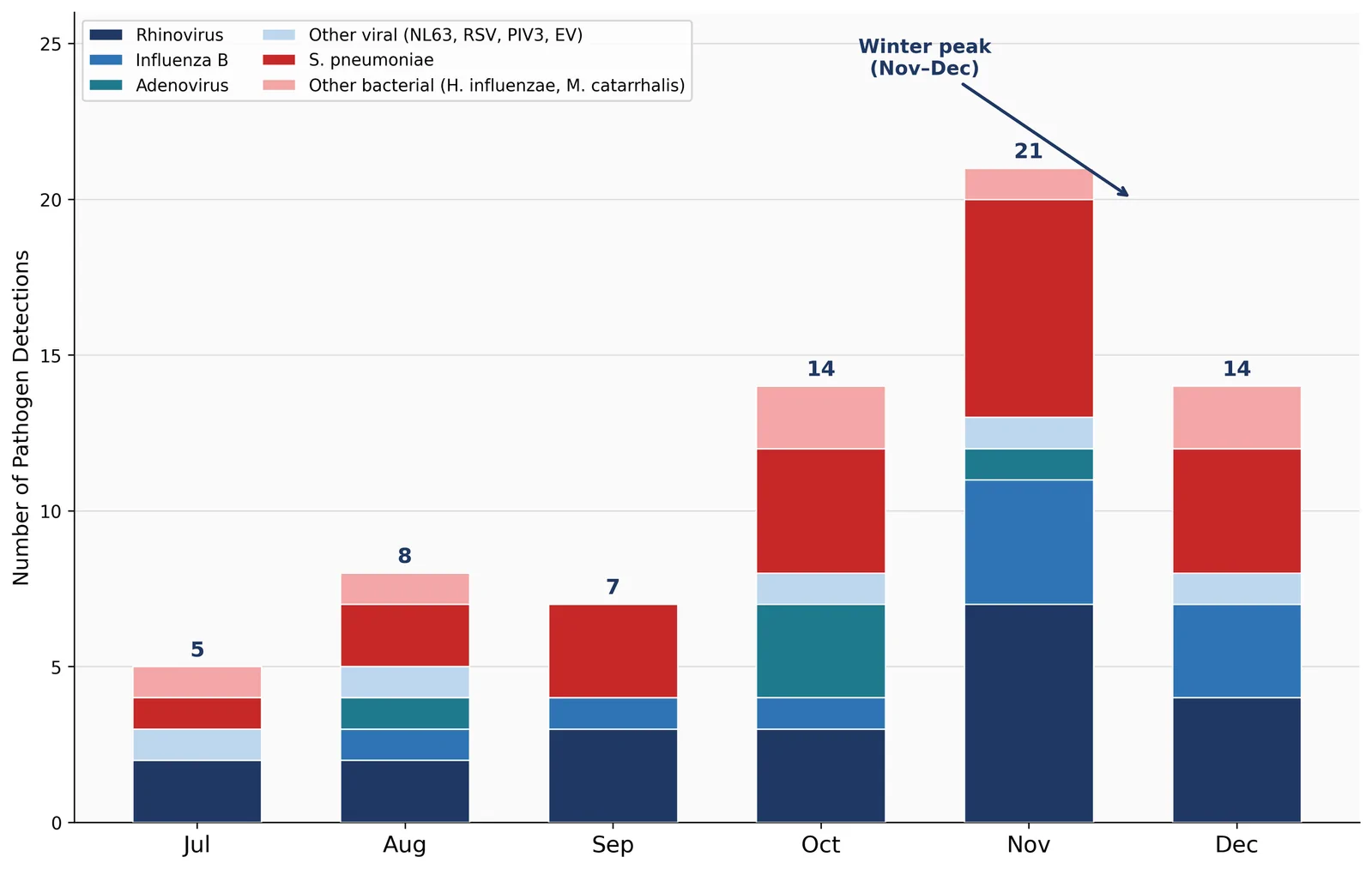
Monthly distribution of pathogen detections (July-December 2022). Rhinovirus and influenza B show a clear winter peak in November-December. *Streptococcus pneumoniae* follows a similar seasonal pattern. Patterns are described descriptively; formal seasonality testing was not performed given the six-month window and small per-month counts.

Rhinovirus and influenza B detections were concentrated in November and December, consistent with the winter respiratory season in Bangladesh. Adenovirus was detected predominantly in September and October. *S. pneumoniae* followed a similar winter peak. Other pathogens showed sporadic, non-clustered detection throughout the six-month period (Figure 7). Given the six-month observation window and small per-month counts, these patterns are reported descriptively; formal seasonality testing was not performed.

## Discussion

The central finding of this study is both clinically important and easy to state: about 36% of adults who tested negative for SARS-CoV-2 at our fever clinic actually harbored a different, identifiable respiratory pathogen. If multiplex PCR had not been performed, most of these infections would have gone undiagnosed. That is the practical case for broad-spectrum respiratory testing in SARS-CoV-2-negative patients with persistent symptoms.

Human rhinovirus dominating the viral findings (45.7% of viral detections; BH p < 0.001) is the most important result of this study. Rhinovirus was not merely common; it was statistically singular, with an adjusted standardized residual of +6.08, appearing more than six SDs above what a uniform distribution would predict. Several features of rhinovirus biology explain its resilience during the pandemic. It is non-enveloped, making it substantially more resistant to the environmental conditions that inactivate SARS-CoV-2 and influenza [11]. Its primary transmission routes include direct contact and indirect fomite transfer, which are less effectively disrupted by droplet-focused interventions such as surgical masking and physical distancing [12]. Its incubation period is short (typically 1-4 days) and its infectious dose low, enabling rapid community spread [13]. A pre-pandemic study at this same institution found rhinovirus ranking third (6.4%) behind coronaviruses and influenza [14]. The near-eightfold increase in relative frequency seen here likely reflects reduced competition from enveloped viruses suppressed by non-pharmaceutical interventions (NPIs), alongside increased testing of a population specifically selected by SARS-CoV-2 negativity.

*S. pneumoniae* dominating bacterial detections (72.4%; BH p < 0.001) is the study’s second major finding. *S. pneumoniae* is a leading cause of community-acquired pneumonia worldwide, and several mechanisms may explain its predominance in this pandemic-era cohort. Viral respiratory infections are well established as facilitators of secondary bacterial invasion, with viral infection disrupting mucosal barrier integrity and modulating innate immune responses [15]. Pandemic conditions may have exacerbated this through delayed healthcare-seeking and altered antibiotic prescribing patterns. These findings reinforce the case for pneumococcal vaccination in Bangladesh, where adult coverage remains suboptimal despite available vaccines [16].

*M. catarrhalis* was significantly underrepresented (BH p = 0.014). This may reflect genuine epidemiological suppression during the pandemic or differential sensitivity of the PCR panel, and we would not overinterpret this finding.

The seasonal concentration of rhinovirus and influenza B in November-December is consistent with documented winter seasonality of these pathogens in South Asia, where cooler temperatures and lower relative humidity enhance aerosol stability and promote mucosal susceptibility [17,18]. *S. pneumoniae* followed a similar temporal pattern, consistent with winter co-peaking of bacterial and viral respiratory pathogens observed in community-based surveillance [19].

For the co-infection analysis, the relevant question is whether specific pathogen pairs co-occur more than expected given their individual prevalences, not whether co-infection types are evenly distributed. One-sided Fisher’s exact test on 2 × 2 contingency tables addresses this question directly and was therefore used; binomial exact tests against a uniform distribution would answer a different, biologically less meaningful question and were not used [20,21].

There are several limitations that should be considered. First, the sample of 165 limits power for inferential analyses, particularly for co-infection results where non-significance after BH-FDR correction partly reflects small numbers. Second, recruitment from a single tertiary care hospital introduces selection bias; the pathogen profile may differ in community or primary care settings, and urban overrepresentation limits generalizability to rural Bangladesh. Third, the VIASURE panel is finite, and pathogens outside its target list could not be detected. Fourth, the cross-sectional design provides no outcome data on clinical severity, illness duration, or antimicrobial use. Fifth, individual-level symptom-pathogen data were not available, precluding symptom-pathogen association testing. Sixth, six months of observation is insufficient to characterize annual seasonality, and the small per-month counts precluded formal seasonality testing. Seventh, PCR-based detection of bacterial pathogens, particularly *S. pneumoniae*, *H. influenzae*, and *M. catarrhalis*, cannot distinguish between active infection and asymptomatic nasopharyngeal colonization, as these organisms are known commensals of the upper respiratory tract. Clinical and microbiological correlation would be required to confirm the pathogenic significance of bacterial detections in individual patients. Finally, urban or rural residence and patient sex could not be formally tested as predictors of pathogen positivity due to sample size constraints.

## Conclusions

Non-SARS-CoV-2 respiratory pathogens were responsible for 35.8% of influenza-like illness in SARS-CoV-2-negative patients attending a tertiary care hospital in Bangladesh during the COVID-19 pandemic. Two pathogens stand out with clear statistical and clinical significance: human rhinovirus, which accounted for almost half of all viral detections and was the single most overrepresented pathogen in the dataset, and *S. pneumoniae*, which dominated bacterial detections. A negative SARS-CoV-2 test should not be the endpoint of etiological investigation in patients with persistent respiratory symptoms. Multiplex PCR testing might be considered as part of routine workup in resource-appropriate settings, and continued investment in pneumococcal vaccination and broad-spectrum respiratory pathogen surveillance remains essential.
